# Adaptation of *Escherichia coli* Biofilm Growth, Morphology, and Mechanical Properties to Substrate Water Content

**DOI:** 10.1021/acsbiomaterials.1c00927

**Published:** 2021-10-21

**Authors:** Ricardo Ziege, Anna-Maria Tsirigoni, Bastien Large, Diego O. Serra, Kerstin G. Blank, Regine Hengge, Peter Fratzl, Cécile M. Bidan

**Affiliations:** † Max Planck Institute of Colloids and Interfaces, 14476 Potsdam, Germany; ‡ Institut für Biologie/Mikrobiologie, Humboldt-Universität zu Berlin, 10115 Berlin, Germany; § Institute of Molecular and Cell Biology, 2000 Rosario, Argentina

**Keywords:** living materials, water content, *Escherichia coli*, biofilm, morphogenesis

## Abstract

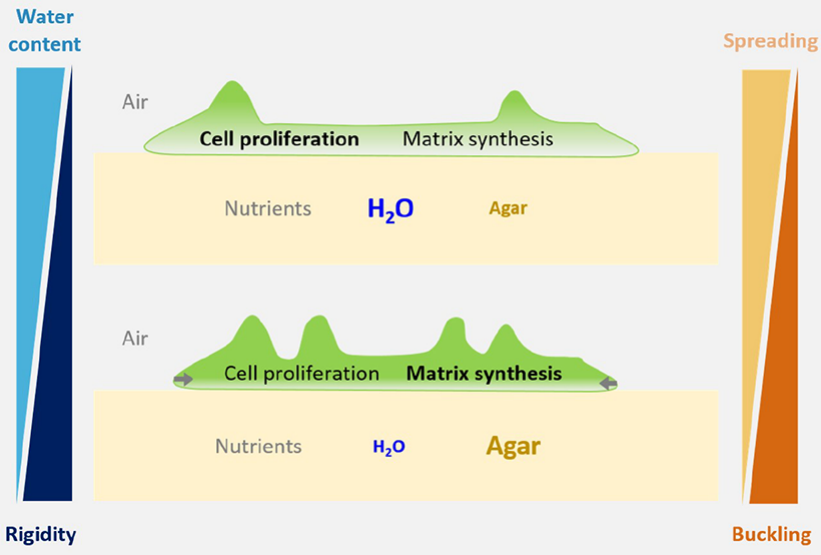

Biofilms are complex living materials that form as bacteria become embedded in a matrix of self-produced protein and polysaccharide fibers. In addition to their traditional association with chronic infections or clogging of pipelines, biofilms currently gain interest as a potential source of functional material. On nutritive hydrogels, micron-sized *Escherichia coli* cells can build centimeter-large biofilms. During this process, bacterial proliferation, matrix production, and water uptake introduce mechanical stresses in the biofilm that are released through the formation of macroscopic delaminated buckles in the third dimension. To clarify how substrate water content could be used to tune biofilm material properties, we quantified *E. coli* biofilm growth, delamination dynamics, and rigidity as a function of water content of the nutritive substrates. Time-lapse microscopy and computational image analysis revealed that softer substrates with high water content promote biofilm spreading kinetics, while stiffer substrates with low water content promote biofilm delamination. The delaminated buckles observed on biofilm cross sections appeared more bent on substrates with high water content, while they tended to be more vertical on substrates with low water content. Both wet and dry biomass, accumulated over 4 days of culture, were larger in biofilms cultured on substrates with high water content, despite extra porosity within the matrix layer. Finally, microindentation analysis revealed that substrates with low water content supported the formation of stiffer biofilms. This study shows that *E. coli* biofilms respond to substrate water content, which might be used for tuning their material properties in view of further applications.

## Introduction

Biofilms are surface attached microbial communities embedded in a self-produced extracellular matrix serving a structural function.^1^ As such, they provide protection to the microorganisms against external stresses, like antibiotics. As the matrix is highly hydrated, biofilms contain more than 80% of water, protecting the encased bacteria from desiccation.^2^ Biofilm growth and mechanical properties are therefore expected to be susceptible to the moisture in their environment. Today, most strategies to engineer living materials from bacteria involve genetic approaches from synthetic biology.^3,4^ While increasing evidence shows how single bacteria respond to external stimuli on a cellular level, it remains largely unknown how external stimuli affect biofilm properties as a whole^5^ and how this knowledge could be leveraged to design biofilm-based functional materials. Here, we explore how tuning the water content in the biofilm substrate can be utilized for tuning biofilm growth, morphology, and mechanical properties.

At a solid–air interface, biofilm growth leads to both lateral spreading and accumulation of internal mechanical stresses, which are introduced by nonuniform growth into increasingly constrained space.^6−8^ To release these internal stresses, flat biofilms buckle toward the third dimension. This leads to complex morphologies, including radial, circumferential, or zigzag wrinkles and delaminated buckles that subsequently emerge in different regions of the biofilm. The development of these surface morphologies was proposed to be governed by (i) a mismatch strain of the biofilm relative to the substrate, induced by biofilm growth; (ii) the ratio of biofilm to substrate stiffness; and (iii) biofilm to substrate adhesion.^9^

The role of substrate water content (i.e., agar concentration) in this interplay of biofilm growth, morphology, and mechanical properties has been studied in biofilm model organisms such as *Bacillus subtilis*(10,11) and *Vibrio cholerae*.^7,12,13^ In the latter case, the bacteria were shown to synthesize matrix macromolecules that establish an osmotic pressure difference between the biofilm and the substrate, leading to water transport, swelling, and thereby enhanced nutrient uptake by the biofilm.^12^ Note that the authors demonstrated the minor role of substrate stiffness on the observed differential biofilm growth behavior by repeating the experiment on identical semipermeable membranes laid on the different agar substrates.

This proposed mechanism of osmotically driven biofilm spreading by swelling of matrix-rich layers is particularly relevant for biofilms formed at a solid–air interface, where water is not in excess but rather slowly driven through the biofilm–substrate interface via diffusion and osmotic pressure gradients.^14^ In addition, evaporation of water from the biofilm–air interface was proposed to enhance nutrient transport through the biofilm in *B. subtilis* biofilms, specifically in highly wrinkled or delaminated regions with a larger surface area.^15^ Substrate water content is also negatively correlated with agar content, of which small variations (in the range of 1%) induce large differences in substrate stiffness,^16^ as well as in surface friction due to the presence of a thin layer of water at the surface.^17^ While not determining for biofilm growth,^12^ such mechanical properties of the substrate can greatly influence biofilm morphology as demonstrated by multilayer theoretical models.^18^

To investigate the relationship between substrate water content and biofilm properties, *Escherichia coli* K-12 AR3110 is used. The *E. coli* strain AR3110 is a W3110 derivative with a restored capacity to produce phosphoethanolamine (pEtN)-modified cellulose.^6,19^ Thus, AR3110 is a highly proficient biofilm-forming strain that produces both amyloid curli protein and pEtN-cellulose as major matrix components. At the solid–air interface, the combination of these two matrix fibers confers AR3110 biofilms with the tissuelike elasticity required to form radial wrinkles at the periphery, which transition to high-aspect-ratio delaminated buckles (height/thickness reaching 10–40).^20,21^ In contrast, macrocolonies grown from the cellulose-deficient strain W3110 present a morphology with thick concentric wrinkles, and no morphological structures are observed for macrocolonies grown from curli- and cellulose-deficient strains. Moreover, matrix-producing *E. coli* AR3110 bacteria encase themselves in a dense network of remarkable microscale architecture in the upper layer of the biofilms (closer to the biofilm–air interface), while bacteria in the bottom layer (closer to the nutritive substrate) do not produce matrix fibers but ensure cohesion through the entanglement of their flagella.^6,19^

In the present work, we explore how *E. coli* K-12 AR3110 biofilms adapt their spreading kinetics, morphology, and rigidity to the water content of the substrate. We first study biofilm spreading kinetics with time-lapse imaging and correlate it with the delamination dynamics and the emerging morphology during and after biofilm growth. We then investigate biomass accumulation and biofilm water content as a function of substrate water content. Finally, we compare biofilm spreading and delamination behavior to their mechanical properties and highlight how these features change as a function of substrate water content.

## Results

### Substrates with High Water Content Promote *E. coli* Biofilm Spreading Kinetics

**Figure 1:**
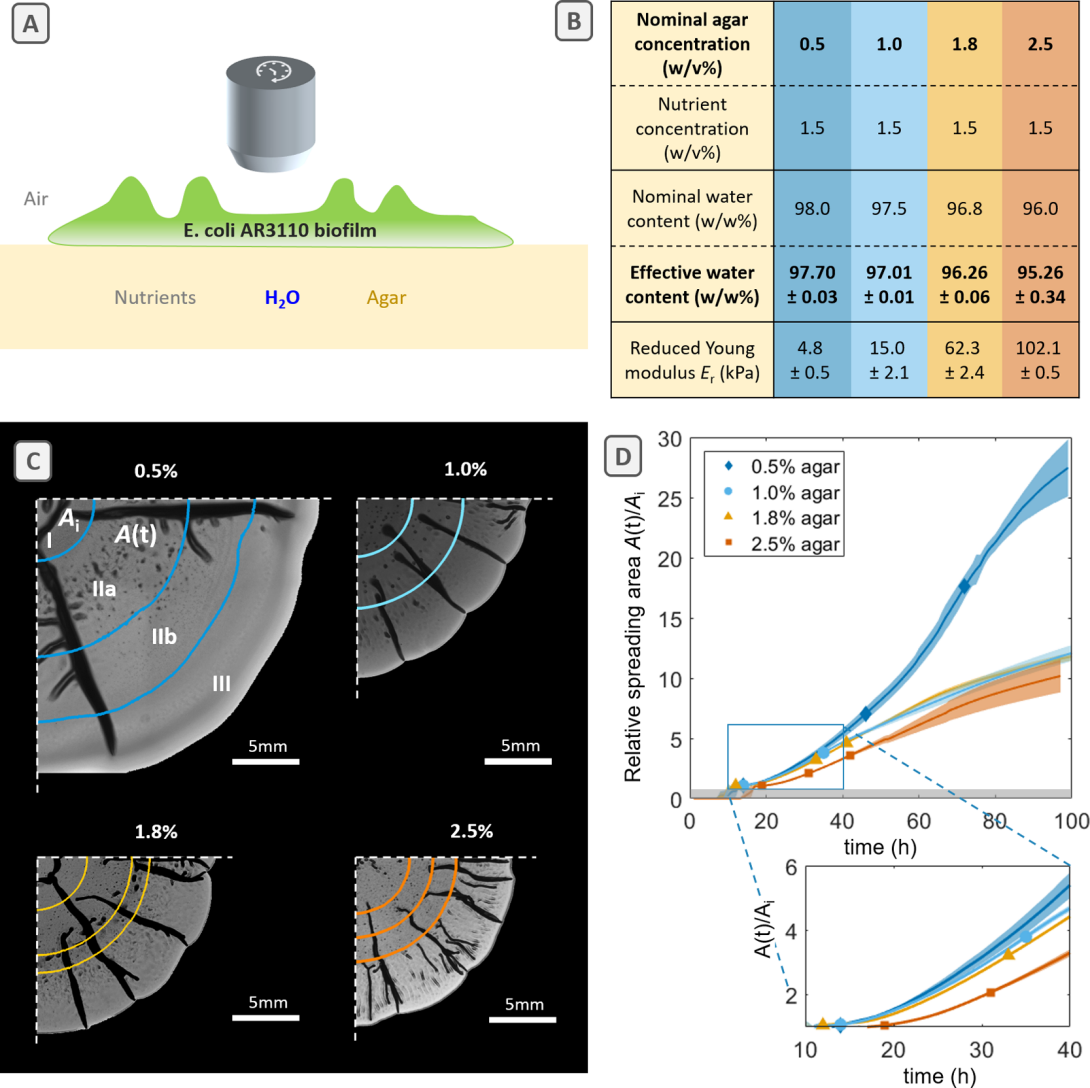
*E. coli* AR3110 biofilm spreading kinetics on nutritive substrates with various agar concentrations. (A) Sketch of the live-imaging setup. (B) Nominal and effective water contents and reduced Young’s moduli *E*_r_, respectively, calculated and/or measured for various nominal agar concentrations supplemented with 1.5% w/v nutrients. (C) Bright-field image of a quarter of the biofilm after 90 h of growth on substrates with the respective agar concentration. Colored outlines delimit the transitions of phases I–II (inner radius), IIa–IIb (middle radius, explanation in next section), and II–III (outer radius). Note that the latter two transitions happen at the same time point (35 h) for biofilms grown on 1.0% agar. (D) Relative spreading area increase during 100 h of growth. The time points of the phase I–II, IIa–IIb, and II–III transitions are indicated (symbols). (D, inset) Zoom-in of the relative area increase between 10 and 40 h of biofilm development. Individual measurements range from *n* = 3 to 9 per condition, and standard deviations are shown as shaded, colored areas.

To understand how *E. coli* biofilm growth is influenced by substrate water content, we first explored their spreading behavior on nutritive, hydrogel substrates of different nominal agar concentrations between 0.5% and 2.5% w/v (Figure 1A). The resulting substrates thus present different effective water contents between 97.70% and 95.26% w/w and distinct mechanical properties ranging from 4.8 to 102.1 kPa as characterized by microindentation (Figure 1B and Figure S1). For this, we inoculated the various agar substrates with 5 μL of *E. coli* AR3110 cell suspensions (∼2.5 × 10^6^ cells/μL) and monitored biofilm growth by time-lapse imaging (Figure 1C). The projected spreading area of the biofilm was measured as a function of time and plotted relative to the initial area *A*_i_, to account for variations of the initial droplet diameters (Figure 1D). To better visualize the spreading kinetics, we further plotted the derivative of the relative area increase *A*(*t*)/*A*_i_ (Supporting Information, Figure S2).

We identified the following 3 phases of *E. coli* biofilm development: In phase I, bacteria remain confined in the circular area defined by the drop of bacteria suspension initially inoculated onto the agar surface (*A*(*t*)/*A*_i_ ≤ 1, Figure 1D). In phase II, biofilms start spreading rapidly in lateral directions (*A*(*t*)/*A*_i_ > 1, Figure 1D) until they reach a maximum spreading rate (Supporting Information, Figure S2). In phase III, biofilm spreading slows down as characterized by a slower increase of relative projected spreading area (inflection of the spreading curves on Figure 1D).

**Figure 2:**
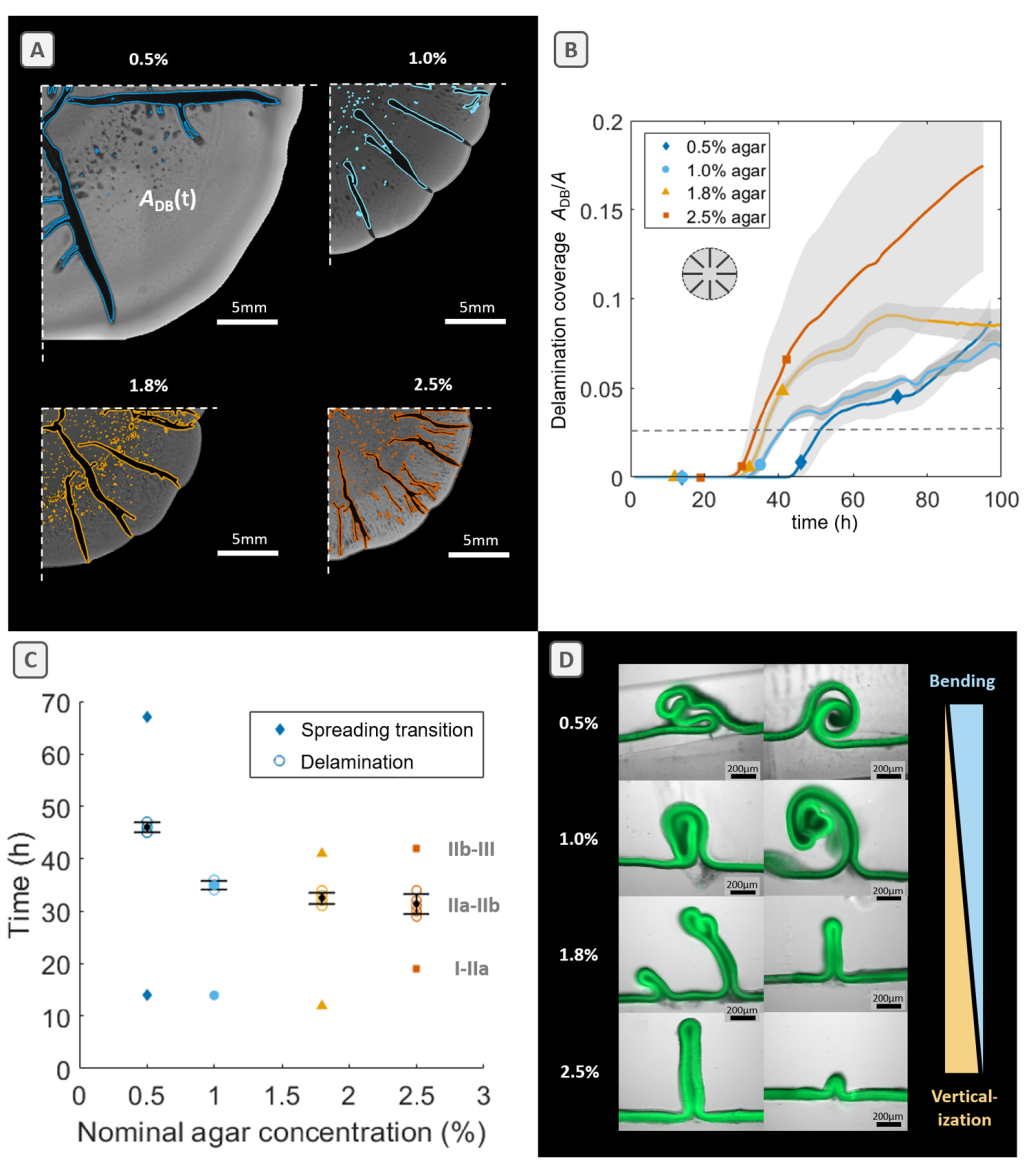
*E. coli* biofilm delamination dynamics and cross-sectional delaminated buckle morphology on nutritive substrates with various agar concentrations. (A) Bright-field images of quarters of individual biofilms grown for 90 h on substrates with the respective agar concentration. Colored outlines represent the delaminated buckle contours at 90 h. (B) 2D projected delamination coverage *A*_DB_/*A*(*t*) during biofilm development on substrates with different water contents. Symbols indicate transitions I–II (first), IIa–IIb (second), and II–III (third). Individual measurements range from *n* = 3 to 9 per condition, and standard deviations are shown as shaded areas. (C) Average onset times of biofilm lateral spreading (bottom, *t* = 12–19 h, I–II), biofilm delamination (middle, *t* = 31–46 h, IIa–IIb), and slow down of spreading (top, 35–67 h, II–III). (D) Bright-field images of cross-sectional cuts of biofilm wrinkles at 100 h of growth. Fluorescence images (green) showing matrix components stained with thioflavin S are overlaid.

While these 3 phases are observed in all conditions, they appear shifted in time. For example, the onset of biofilm spreading, which defines the beginning of phase II, appeared at later time points on substrates with low water content (Figures 1D and 2C, 0.5% agar). Indeed, biofilms grown on substrates with high and medium water content (0.5%, 1.0%, and also 1.8% agar) started expanding laterally after 12–14 h. In contrast, biofilms grown on substrates with low water content (2.5% agar) initiated spreading on average 19 h after inoculation (Figure 2D, inset).

Moreover, the relative projected spreading area *A*(*t*)/*A*_i_ increased faster on substrates with high water content (Figure 1D). 20 h after entering phase II, the average relative spreading area of biofilms grown on 0.5% agar increased up to 4-fold when compared to the area of the initial drop, whereas it expanded only 3-fold in the case of biofilms grown on 2.5% agar (Figure 1D, inset). This trend continued until 70 h after the onset of phase II. Biofilms grown on 0.5% agar showed a 23-fold increase in spreading area, whereas biofilms grown on 2.5% agar only reached an average area increase of 9.5-fold (Figure 1D).

Biofilms grown on agar of medium and low water content (1.0–2.5% agar) displayed an initial accelerated spreading to reach a maximum spreading rate and entered phase III between 35 and 42 h (Figure 1D; Supporting Information, Figure S2). Interestingly, biofilms grown on 1.0% and 1.8% agar showed similar spreading kinetics in their phase III, while biofilms grown on substrates with high water content (0.5% agar) exhibited an accelerated spreading until the late stage of biofilm development and only entered phase III after 67 h (Supporting Information, Figure S2).

### Substrates with Low Water Content Increase Biofilm Buckling

As biofilms grow on a two-dimensional surface and accumulate biomass and subsequently internal mechanical stresses, they eventually bend in the third dimension in the form of wrinkles and eventually delaminated buckles (Figure 1C). We thus asked if and how the observed effect of water content on biofilm spreading relates to the emergence of long and radial delaminated buckles (Supporting Information). To compare the dynamics of this buckling process for different water contents, we measured the ratio of delaminated buckle-to-biofilm projected area as a function of time *A*_DB_(*t*)/*A*(*t*) (Figure 2A,B). While biofilm growth cannot be fully characterized in 3D with our system, this parameter called “projected delamination coverage” allows estimating quantitatively the ratio of biofilm growing in the vertical direction.

We first observed a delayed onset of delamination for biofilms grown on substrates with high water content (Figure 2B, dark blue line plot) and defined the onset of buckling as the transition between phases IIa and IIb (Figure 2C). Biofilms grown on 0.5% agar substrates showed a delamination coverage >2.5% only after 51 h. The first wrinkles appeared at about 20 h before their entry into phase III. In contrast, biofilms grown on 1.0%, 1.8%, and 2.5% agar substrates displayed a delamination coverage of >2.5% already after 41, 36, and 34 h, respectively. This is less than 10 h before entering the decelerating phase III of biofilm spreading for biofilms grown on 1.8% and 2.5% agar, whereas the onset of buckling and the transition to phase III coincided for biofilms grown on 1.0% agar. Interestingly, the onset of buckling corresponds to a slight decrease of spreading acceleration in all of the conditions, but the latter is only temporary in the case of biofilms grown on 0.5% agar substrates (Supporting Information, Figure S2).

Figure 2B also reveals a larger coverage with delaminated buckles for biofilms grown on substrates with low water content. After 90 h, biofilms grown on 2.5% agar substrates were covered with up to 20% of delaminated buckles, whereas biofilms grown on wetter substrates only reached an average delamination coverage of maximally 7–8% (Figure 2B). The values are surprisingly similar for biofilms grown on 0.5%, 1.0%, and 1.8% agar substrates, considering that their buckling history is different.

The above results suggest that biofilms grown on substrates with high water content mainly rely on two-dimensional spreading while biofilms grown on dryer substrates mainly rely on the formation of three-dimensional delaminated buckles to distribute their biomass made of bacteria and hydrated matrix. We thus explored the morphology of the delaminated buckles as a function of biofilm growth conditions in more detail. As evidenced in Figure 2A, the delaminated buckles formed on biofilms grown on high-water-content substrates have a larger projected width when compared to delaminations formed on biofilms grown on substrates with low water content. To explain this observation, we embedded biofilms grown on the various substrates of interest in agar blocks and prepared cross sections of the buckled regions (periphery). The resulting images reveal a nearly constant biofilm peripheral thickness, which ranges from 60 to 65 μm for biofilms grown on substrates with high and medium water content and a slight increase of 80 μm for biofilms grown on substrates of low water content (Supporting Information, Figures S4 and S5). The larger projected width observed for biofilm delaminated buckles formed on substrates with high water content (Figure 2A) originates from a stronger tendency of the delaminated buckles to bend or collapse (Figure 2D). On substrates with low water content, the delaminated buckles form more vertically with a higher aspect ratio, in agreement with what has been described before for AR3110 grown on 1.8% agar.^6^ Interestingly, the delamination process implies that the two bottom sides of the biofilm come in contact and adhere to form mm-scale structures from μm thick biofilms.^8^ For *E. coli* AR3110, the two folded upper layers have been shown to be separated by an area filled with non-matrix-producing cells,^6^ which appears as a gray region in between the two matrix layers in the overlaid images (Figure 2D, 1.0–2.5%). However, the adhesion of these two upper, matrix-rich layers seems to be compromised on biofilms grown on substrates with high water content, as suggested by the white zones in the bright-field image between two detached matrix-rich layers in the overlaid images (Figure 2D, 0.5%). Note that we cannot completely exclude that this is as an artifact of our cross-sectioning protocol, though the same morphologies were observed on several cross-sectioned delaminated buckles.

The different dynamics and apparent stabilities of the delaminated buckles, observed for *E. coli* AR3110 biofilms grown on substrates with various water contents, suggest that the biofilm composition and/or matrix distribution are affected by the water availability from the substrate.

### Substrate Water Content Affects Biofilm Weight, Water Content, and Matrix Distribution

*E. coli* biofilm growth at a solid–air interface is expected to result from a combination of cell proliferation, matrix production, and water uptake. The total wet mass of a biofilm *m*_w_ comprises the mass fractions of cells ϕ_cells_, matrix ϕ_matrix_, and water ϕ_water_ so that ϕ_cells_ + ϕ_matrix_ + ϕ_water_ = 1.^22^ (Note that this theoretical study of Srinivasan et al. used volume fractions, which are harder to measure experimentally due to the limited access to three-dimensional quantitative information.) To assess the respective contributions of these components, we grew biofilms on substrates of different water contents for 4 days, scraped them from the agar surfaces, measured their wet (*m*_w_) and dry (*m*_d_) mass, and calculated the biofilm effective water contents *W* = ϕ_water_ × 100% = (*m*_w_ – *m*_d_)/*m*_w_ × 100% w/w.

**Figure 3:**
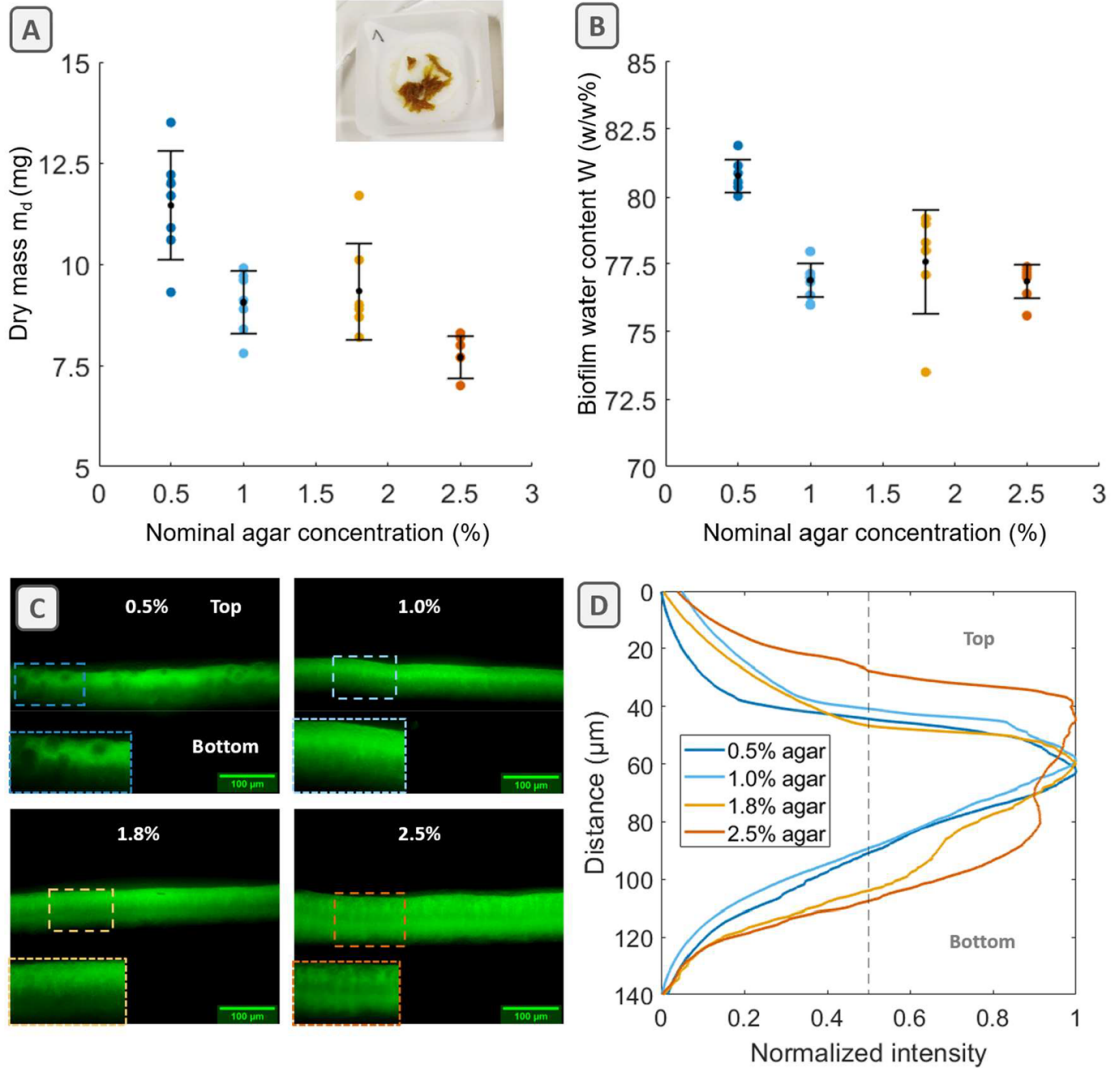
Dry mass, water content, and matrix distribution of *E. coli* biofilms grown on nutritive substrates with various agar concentrations. (A) Average dry masses *m*_d_ and (B) effective water content *W* of single biofilms grown for 4 days. (C) Fluorescence images of *E. coli* AR3110 peripheral biofilm cross sections, depicting the distribution of amyloid curli protein and pEtN-modified cellulose fibers stained with thioflavin S (green fluorescence). (D) Normalized average intensity profiles recorded over each biofilm cross-sectional area as indicated in (C) the rectangular color-coded zoom in. Full width at half-maximum (fwhm) is indicated as a dashed line at 0.5, showing increased thickness of the matrix layer in biofilms grown on 2.5% agar. For wet and dry mass as well as water content measurements, *n* = 7 individual biofilms per condition. Error bars indicate one standard deviation.

*E. coli* AR3110 biofilms grown on substrates with high water content (0.5% agar) produced on average 1.8 times the amount of wet mass *m*_w_ relative to substrates with low water content (2.5% agar) (Supporting Information, Figure S3). Upon drying at 60 °C for 3 h, single biofilms become fully dehydrated due to their small volumes, and the remaining dry biomass *m*_d_ contains bacteria embedded in extracellular matrix components. This drying procedure yielded brittle yet intact pieces of biofilm material (Figure 3A, inset). Interestingly, biofilms grown on substrates with high water content did not only contain a higher wet mass than biofilms grown on low-water-content substrates. On average, they also yielded 1.7 times more dry mass (Figure 3A). Indeed, the total dry mass weighed 11.5 mg for biofilms grown on substrates with high water content (0.5% agar), and the dry mass decreased with decreasing water content to 7.7 mg on 2.5% agar (Figure 3A).

As we observed a slight change in the ratio of dry to wet mass with agar concentration, we calculated biofilm effective water contents gravimetrically. Figure 3B shows that the water content of the biofilms (*W*) increased as the agar concentration of the substrate decreased. Biofilms grown on substrates with high water content contained on average 80.8% water, whereas biofilms grown on low-water-content substrates stored on average 76.9% water. This indicates a change in the fraction of biomass (ϕ_cells_ + ϕ_matrix_) inside the biofilm, which in turn suggests a change in the composition of the biofilm that may be accompanied by changes in matrix distribution. To verify this hypothesis, we analyzed cross sections obtained from the periphery of biofilms grown on the various substrates. Figure 3C shows cross-sectional images of single biofilms where the matrix components amyloid curli and pEtN-cellulose fluorescently were again stained with thioflavin S.

For *E. coli* AR3110 macrocolonies, the production of matrix components has been shown to be asymmetric with curli and pEtN-cellulose being synthesized exclusively in the upper layer exposed to air.^23^ Within this layer, subzones that exhibit homogeneous or heterogeneous production of curli and/or pEtN-cellulose were also distinguished.^24^ For biofilms grown on 1.8% agar, we observed this asymmetric distribution of matrix components as reported before (Figure 3C,D). Note that the 10 μm bottom layer of highly flagellated and non-matrix-producing cells was probably lost during sample preparation. By lowering the substrate water content (2.5% agar), we find a more symmetric distribution of matrix components from the top to the bottom of the biofilm (Figure 3C,D), whereas the asymmetry seemed to be preserved for the biofilm grown on 1.0% agar (Figure 3C,D). Interestingly, biofilms grown on substrates with high water content (0.5% agar) also revealed this asymmetric distribution of matrix components (Figure 3C,D) and presented additional heterogeneities penetrating the thick matrix layer at the top of the biofilm, which probably contains regions of non-matrix-producing cells that are not stained by thioflavin S. Clearly, the distribution of matrix across the biofilm cross section changes depending on substrate water content while the biofilm thicknesses range from 60 to 80 μm for biofilms grown on substrates with high and medium water contents and up to 80–100 μm for biofilms grown on substrates with low water content (verified from bright-field images of peripheral and central cross sections; Supporting Information, Figures S4 and S5). Indeed, thicker matrix-rich layers are formed on biofilms grown on substrates with low water content (2.5% agar), whereas thinner and more porous matrix-rich layers are formed on substrates with high water content (0.5% agar) (Figure 3C,D).

### Biofilms Are Stiffer When Grown on Substrates with Low Water Content

**Figure 4:**
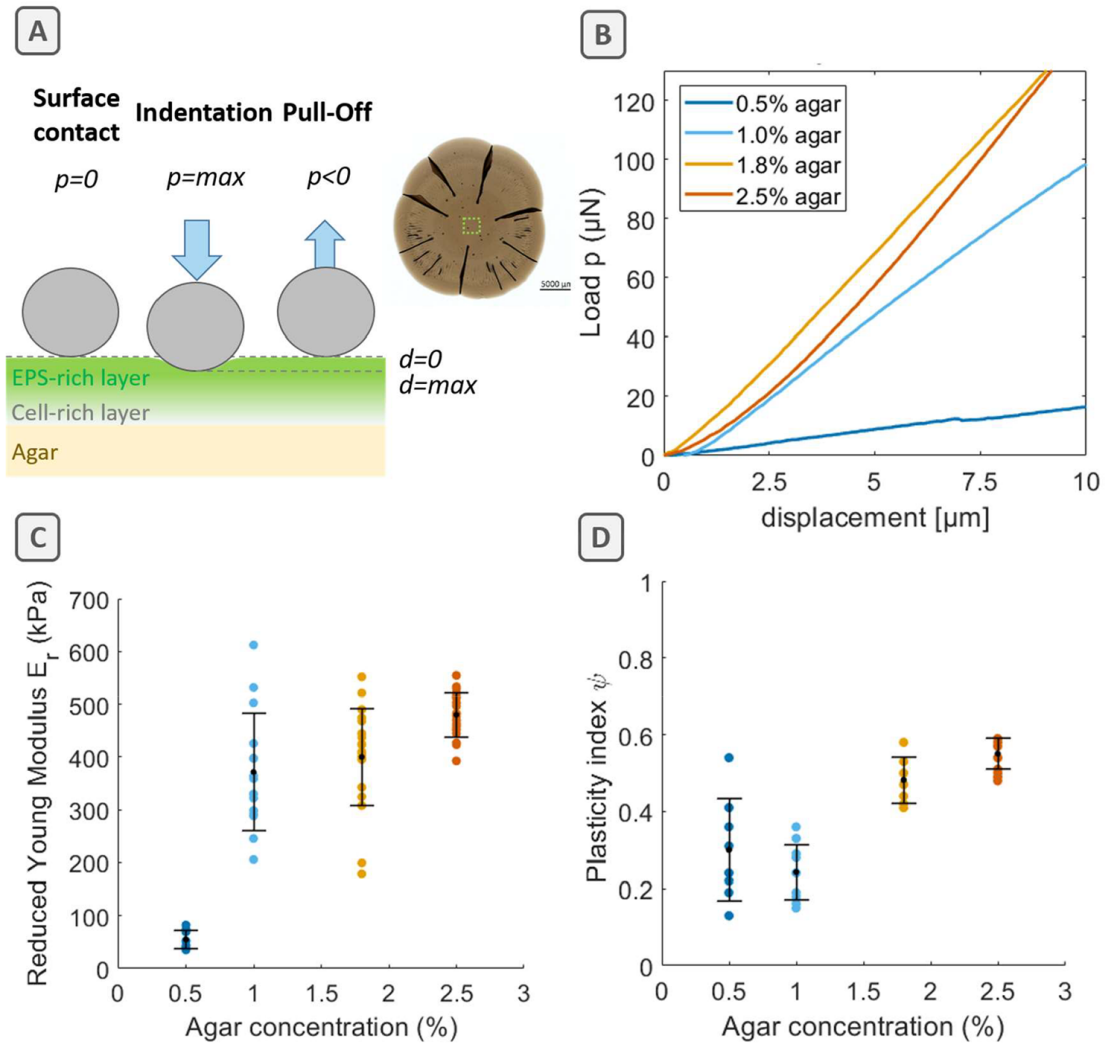
Microindentation of *E. coli* biofilms grown on nutritive substrates with various agar concentrations. (A) Sketch of surface indentation during loading and unloading the biofilm surface. (B) Load–displacement curves when indenting the biofilm surface (loading curve). (C) Averaged reduced Young’s modulus *E*_r_ values, describing the measured rigidity of the biofilm surface. (D) Averaged plasticity indices ψ, describing the ratio between dissipated (1 = fully irreversible) to elastically stored (0 = fully reversible) energy during indenting the biofilm surface. The number of individual measurements is *n* = 8–23 per condition. Shown are mean values and standard deviations as error bars.

As the water content and composition of biogenic viscoelastic materials are key determinants for their mechanical performance, we hypothesized a further impact of the water content of the substrate on the mechanical properties of the biofilms. To assess how the observed differences in biofilm composition and matrix distribution translate into their mechanical properties, especially their rigidity or Young’s modulus *E*, we performed microindentation experiments in the biofilm center (Figure 4A, photo). Upon contacting the biofilm surface with a spherical diamond tip of radius *R* = 50 μm, the biofilms were indented by 7–30 μm. This allowed for locally probing the biofilm material in compression over a contact area that encompasses several bacteria embedded in a dense matrix, according to previous descriptions of the top layer of *E. coli* AR3110 biofilms.^6^

To estimate the rigidity of the biofilm, we calculated the reduced Young’s modulus values *E*_r_ from the load–displacement curves. We assumed a linear elastic response of the material at small deformations, i.e., at indentation depths of approximately 1/10 of the biofilm thickness, which is considered to measure approximately 100 μm in the central region. We then fitted a Hertz model to the load–displacement curves, ranging from the contact of the tip on the surface (0 μm) to an indentation depth of maximum 10 μm (Figure 4B). As expected, the resulting reduced Young’s moduli *E*_r_ varied with the agar concentration of the substrate. Indeed, biofilms were 1 order of magnitude stiffer when grown on substrates with low water content, following a nonlinear increase of reduced Young’s moduli *E*_r_ from 50 kPa for biofilms grown on substrates with high water content to 360, 400, and 500 kPa for biofilms grown on 1.0%, 1.8%, and 2.5% agar substrates, respectively (Figure 4C).

We further observed a hysteresis behavior between the loading and unloading curves. This allowed us to derive a plasticity index ψ, which estimates the reversibility of the surface deformation.^25^ The plasticity index ψ, calculated from the areas under the loading and unloading curves, compares the amounts of energy stored (elastic behavior) and dissipated (viscous and plastic behavior) during the deformation of the biofilm (Figure 4D; Supporting Information, Figure S6). Despite substantial differences in their rigidity (*E*_r_), the ratio of elastic vs plastic deformation appears similar (around 0.5) for biofilms grown on substrates with low and medium water contents (1.8% and 2.5% agar). In contrast, biofilms grown on substrates with high and medium water contents (0.5% and 1.0% agar) present a lower plasticity index around 0.2–0.3. All in all, these results suggest that both the rigidity of the biofilm material as well as the energy elastically stored adapt to the water content of their nutritive substrate.

## Discussion

**Figure 5:**
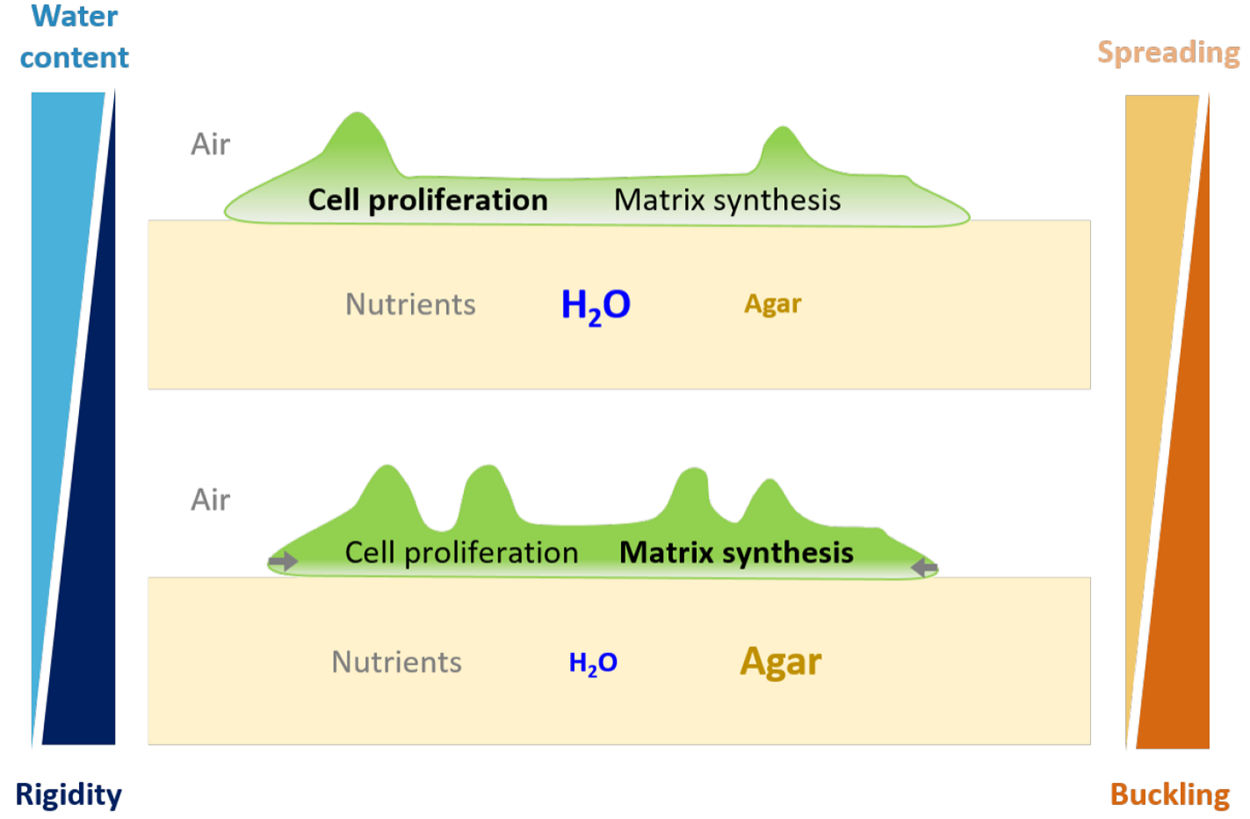
*E. coli* AR3110 biofilms have higher water content when grown on substrates with high water content (top) while they are more rigid when grown on substrates with low water content (bottom). This is consistent with the higher cell proliferation expected to be supported by a nutrient supply facilitated on substrates with high water content and with the observation of a more densely and homogeneously distributed matrix across biofilms grown on substrates with low water content. Together with these biofilm material properties, the interfacial friction at the surface of the agar may largely contribute to various morphologies of *E. coli* AR3110 biofilms as they spread more on substrates with high water content (top) while they tend to grow in the third dimension on substrates with low water content (bottom).

While previous studies of *E. coli* biofilm development and the emergence of complex structures focused on genetically driven effects resulting from nutrient and metabolic gradients,^23^ the present work explores the interplay of these morphological features with *E. coli* biofilm mechanics and puts a particular focus on the influence of the water content in the environment. Specifically, we have demonstrated that *E. coli* AR3110 biofilms, grown at the solid–air interface, adapt their spreading kinetics, morphology, and mechanical properties to the water content of the agar substrate (Figure 5). In comparison, the morphogenesis of *B. subtilis* and *V. cholera* biofilms has already been shown to be influenced by agar concentration (i.e., water content). These biofilms adopt faster spreading kinetics on wet substrates and develop more morphological features on dryer substrates.^7,10−13^ Here, we show that *E. coli* AR3110 biofilms adopt a similar behavior (Figures 1A and 2A), which infers that similar physical mechanisms are involved, namely, surface forces,^26^ water evaporation, and osmotic swelling, rather than microbial sensing of substrate stiffness.^12^

*E. coli* biofilm spreading on wet substrates may be facilitated by the reduction of interfacial friction (or tangential adhesion) between the biofilm and the substrate due to a thin layer of water directly available at the surface.^8,17^ Note that, in extreme cases, like on 0.5% agar, one approaches the concentrations used for *E. coli* swimmer plates so that such conditions might also promote bacterial swimming motility favorable to biofilm spreading.^27^ On dryer substrates, however, interfacial friction was proposed to constrain biofilm spreading mechanically, thereby causing a continuous compression of the biofilms as they grow.^7,8^ The accumulation of such tangential compressive stresses further leads to mechanical instabilities like buckling events from which surface wrinkles emerge. Once the normal adhesion forces on the agar gel are overcome, the biofilm delaminates from the substrate and further grows in the third dimension. Delayed biofilm spreading and early wrinkling and delamination, as observed on dryer substrates (Figure 2C), suggest that friction plays a similar role here. Inversely, delayed buckling in biofilms grown on substrates with high water content may be attributed to the early and large spreading rates, which may slow down the accumulation of compressive stresses. Note that substrates with high water content have a higher compliance to the deformations induced by biofilm growth (Figure 1B and Figure S1), but these are likely screened by the reduced adhesion and friction forces resulting from the abundance of water at the surface, as suggested by the few but large delaminated buckles (Figure 2D). Interestingly, enhanced buckling of *E. coli* AR3110 biofilms due to confined growth has recently been obtained independently of substrate stiffness by coating the agar surface with positively charged polyelectrolytes.^28^ In this context, interfacial friction was proposed to result from physicochemical interactions between negatively charged bacteria and positively charged coatings.

As demonstrated for *B. subtilis* biofilms, buckling also increases the surface area at the biofilm–air interface and promotes the evaporation of water.^15^ This phenomenon therefore constitutes a potential driving force to transport nutrient-carrying water from the substrate to the biofilm and can be particularly useful in conditions of low nutrients and/or water availability. Note that our results obtained with *E. coli* AR3110 are consistent with this proposition as biofilms grown on dryer substrates show larger delamination coverages (Figure 2A,B).

Matrix swelling is another mechanism proposed to be involved in the adaptation of biofilm morphology to substrate water content.^12,14,26^ Indeed, the excretion of matrix components by the bacteria creates osmotic gradients, inducing water uptake and biofilm swelling. At the interface with substrates of low agar concentrations (i.e., high water content), such gradients are expected to be particularly sharp, and the biofilms are expected to take up more water, as we measured in *E. coli* AR3110 biofilms (Figure 3B). For *E. coli* AR3110 biofilms, the pEtN-modified cellulosic component is expected to greatly contribute to water-binding and matrix swelling.^29^ This is different for *B. subtilis* biofilms, where water-binding was related to the presence of solutes instead of matrix components.^30^ Together with the lower friction, higher matrix swelling may also partially contribute to the larger biofilm spreading observed on low-agar substrates (Figure 1C,D).

Larger spreading provides a higher number of bacteria with the advantage to be in direct contact with the nutrient-rich surface.^12^ In such favorable conditions, bacteria are expected to favor proliferation upon matrix production,^23^ which could explain the lower matrix signal (Figure 3C) and the lower mechanical properties (Figure 4C) observed for biofilms grown on 0.5% agar substrates, despite their higher dry mass (Figure 3A). Biofilm spreading was observed to slow down later on the substrates with high water content (beginning of phase III, Figure 2C and Figur S2). This change of spreading behavior cannot be explained by the growth in the third dimension alone since the onset of wrinkling and delamination appears earlier in most of the conditions (Figure 2C). Alternative explanations could be a reduction in nutrient supply or matrix swelling and/or an increase of interfacial friction as water from the agar surface diffuses to the biofilms. Further investigations are yet needed to understand the limits of *E. coli* AR3110 biofilm spreading at the solid–air interface.

Besides influencing biofilm morphogenesis, the water content of the underlying substrate also influences the quantity and the quality of the biofilm material produced by *E. coli* AR3110 (Figures 3 and 4). Indeed, biofilms grown on wet substrates contain more water and, at the same time, also a higher dry biomass (Figure 3A,B). These results are consistent with the trend observed on wet masses of *V. cholerae* biofilms measured on substrates with various agar contents and further support the role of osmotic spreading^12^ (Supporting Information, Figure S3). Moreover, a qualitative characterization of biofilm composition using fluorescence imaging indicated that the thickness of the matrix-rich layer as well as the matrix density—thus, the contribution of matrix ϕ_matrix_ to the fraction of biomass (ϕ_cells_ + ϕ_matrix_)—are larger on dryer substrates (Figure 3C). We similarly infer that the contribution of bacteria mass ϕ_cells_ is larger in biofilms grown on wet substrates, which show a thinner matrix-rich layer and lower matrix density. Consistently, microindentation revealed that the reduced Young’s moduli E_r_ of biofilms grown on wet substrates are lower on wet substrates (Figure 4C), where the biofilms contain more water and less matrix, and form delaminated buckles that are mechanically unstable (Figure 2D).

The different rigidities of the *E. coli* AR3110 biofilms thus partially stem from the different compositions of the biofilms (ϕ_cells_, ϕ_matrix_, and ϕ_water_) obtained on the different substrates. In this regard, theoretical models relating polymer volume fractions to osmotic pressure and elastic properties, and which are traditionally used to predict the mechanical behavior of hydrogels, have already been applied to biofilms.^12,31,32^ However, *E. coli* AR3110 biofilms are also known for their asymmetric architecture, which is greatly heterogeneous across their thickness^6,20,23^ and is expected to significantly contribute to the mechanical behavior of the biofilm material. In that regard, *E. coli* AR3110 biofilm morphogenesis may be better described by trilayer models similar to those used for *V. cholerae*,^7^ where the top layer would correspond to the matrix-rich layer of the biofilm; the bottom layer would be the substrate, and the middle layer would be essentially made of water and bacteria (Figure 3D and Figure S3).^20^ In general, applying simple theoretical models on such materials may require approximations that should be considered with precautions, as done with the Hertz model used to estimate the reduced modulus from the microindentation curves obtained in the matrix-rich upper layer of the biofilms (Figure 4A,B). Moreover, both swelling and mechanical properties of a hydrogel strongly depend on the interactions between the macromolecules inside the polymer network. In *E. coli* AR3110 biofilms, the presence of amyloid curli and pEtN-cellulose matrix fibers and their interactions in the form of cross-linking or simple entanglement contribute to the global mechanical behavior.^21^ A greater swelling and lower rigidity of biofilms grown on high-water-content substrates could therefore result from weaker interactions between the matrix fibers due to the larger proportion of water. Even though multiple factors may contribute to biofilm rigidity, the general trend shows that biofilms with lower water content are more rigid. Further studies are yet needed to elucidate how water interacts with the *E. coli* AR3110 biofilm matrix on a molecular level and how this translates into altered mechanical properties.

## Conclusion

Taken together, these structural and mechanical observations are consistent not only with the role of matrix swelling in biofilm spreading (Figure 1) but also with the different wrinkling and delamination behavior observed in biofilms grown on substrates with various water contents (Figure 2). In addition to interfacial friction, nonuniform growth, and substrate stiffness, the buckling behavior of a biofilm is known to depend on its effective mechanical properties,^33^ which in turn depend on its composition and internal structure. Interestingly, the macromorphology of *E. coli* AR3110 biofilms appears to be comparable to the morphology of *B. subtilis* and *V. cholerae* biofilms all characterized by the emergence of long radial wrinkles and delaminated buckles (Figure 1A).^7,8,12,15,34^ However, recent work comparing various mutants of matrix-producing *E. coli* reported distinct micromorphologies,^19^ thereby illustrating the crucial role of the composite nature of the matrix in biofilm morphogenesis. Further dynamic and quantitative studies of biofilm mechanics are required to verify in which conditions the mechanisms proposed for other biofilm-forming bacterial species would apply for *E. coli*.

While the observed adaptation of biofilm material properties to substrate water content certainly provides bacteria with suitable protection against stresses like starvation and desiccation,^20^ it can also be leveraged in the perspective of engineering biofilm-based materials. Indeed, our work points out alternatives to complex synthetic biology and genetic engineering of bacteria for tuning biofilm properties in view of growing functional living materials. Namely, engineering the environment during biofilm production can also yield a large range of material properties. Ultimately, understanding the genetic and biochemical pathways as well as the physicochemical mechanisms involved in the biofilm response to environmental conditions will enable a prediction of the properties of the resulting biofilm-based material. The present study constitutes a first step toward understanding the physicochemical processes. It shows that *E. coli* AR3110 biofilms adapt to the water content of their substrates and contain more water and dry mass when grown on wet substrates. This in turn promotes their spreading area but results in a softer material. In contrast, *E. coli* AR3110 biofilms grown on dryer substrates cover a smaller area and have a denser matrix, which confers them mechanical properties approaching those of mammalian tissues with Young’s moduli of several hundred kPa (*E* ∼ 4*E*_r_/3, Figure 4C). These results are particularly interesting when considering the potential of biofilm-based materials, and especially *E. coli* matrix-based materials for therapeutic applications^35^ and tissue engineering.^36^

## Methods

### Bacterial Strain and Growth

*E. coli* K-12 AR3110 with the repaired synthesis of cellulose components was used throughout this study as the biofilm-forming bacterial strain.^6^ Salt-free agar plates (15 mm diameter) were prepared with 0.5%, 1.0%, 1.8%, or 2.5% w/v of bacteriological grade agar–agar (Roth, 2266), supplemented with 1% w/v tryptone (Roth, 8952) and 0.5% w/v yeast extract (Roth, 2363). Each plate was inoculated with arrays of 4 or 9 drops of 5 μL of bacterial suspension (OD_600_ ∼5.0). The suspension was prepared from a single bacterial colony and grown overnight in Luria–Bertani (LB) medium at 37 °C with shaking at 250 rpm. After inoculation, the excess of water evaporated from the drops and left bacteria-rich disks of comparable sizes from 4 to 8 mm diameter, depending on the growth condition. If matrix staining was needed, thioflavin S (Merck, T1892; 2 mg/mL in 70% ethanol) was added to the liquid salt-free agar directly before pouring to reach a final concentration of 40 μg/mL. Biofilms for live imaging were grown for 100 h at 28 °C in static conditions. Biofilms used for estimation of mass, water content, and mechanical parameters were grown for 4 days in total (∼96 h).

### Gravimetric Water Content and Biomass Measurements

The nominal water contents of the nutritive agar substrates were determined from the respective agar masses used during preparation as *W*_nominal_ = *m*_water_/(*m*_water_ + *m*_agar_ + *m*_nutrients_) × 100% w/w. Their effective water contents were determined gravimetrically by weighing and drying 2 × 2 cm agar gel pieces at 60 °C for 20 h in an oven and calculated from the wet and dry masses (m_wet_, m_dry_) as *W* = (*m*_w_ – *m*_d_)/*m*_w_ × 100% w/w. Effective agar water contents were averaged from 4 independent measurements per condition. The difference in nominal and effective agar water contents results from nutrients being dissolved in the water phase but still contributing to the dry mass measurements.

For biofilm weight and biofilm water content measurements, 7 individual biofilms per condition were scraped from the respective agar substrates after 4 days (∼96 h) of growth. Single biofilms were weighed in weighing boats and dried at 60 °C for 3 h. Wet and dry masses (*m*_w_, *m*_d_) were determined before and after drying. Gravimetric biofilm water contents were calculated as *W* = (*m*_wet_ – *m*_dry_)/*m*_wet_ × 100% w/w.

### Biofilm Imaging and Analysis

For live imaging, biofilms were grown in a custom-made on-stage incubator installed on the motorized stage of an AxioZoomV.16 stereomicroscope (Zeiss, Germany). 3 × 3 and 2 × 2 tile regions were automatically recorded at 4–9 positions on a 15 cm Petri dish over the course of 100 h of biofilm development with 1 h intervals. Temperature and relative humidity inside the on-stage incubator were controlled and set to 28 °C and >90%, respectively.

Biofilm spreading area *A*(*t*) and delaminated buckle area *A*_DB_(*t*) were analyzed automatically, using custom-written MATLAB codes (Matlab 9.7.0 R2019b, MathWorks, Natick, MA). In a first step, thresholding was applied to the intensity images to segment biofilm and wrinkle areas from the background. As image contrast increased due to biofilm growth, thresholding was possible from *t* > 10 h. For each condition, *A*_i_ = 1 was defined at the time point, when all different samples grown in this condition could be detected, to have a common reference point for the calculation of the relative area increase *A*(*t*)/*A*_i_. Onset times of biofilm spreading (transition from phase I to II) were defined as *A*(*t*)/*A*_i_ > 1.05. The transition time point from phase II to III was defined at the maximum of the areal spreading rate 1/*A*_i_ × d*A*/d*t* (Supporting Information, Figure S2). Finally, phase II was later split into phases IIa and IIb, defining the onset of delamination as *A*_DB_(*t*)/*A*(*t*) > 0.005.

### Cross-Sectioning of Biofilms

The protocol established to obtain cross sections of living biofilms was adapted from Figure S7.^37^ The biofilms of interest were isolated by trimming the underlying agar substrate into ∼4 × 4 cm pieces. One piece was placed in a 6 cm diameter Petri dish and slowly but continuously submerged with 50 °C hot liquid salt-free agar (Figure S6B, 1.8%, without supplemented nutrients) while avoiding direct pouring on the biofilm and especially on the delaminated buckles. The resulting agar–biofilm–agar sandwiches were left to solidify for at least 20 min and then further trimmed. With a scalpel, sandwiches were cut to ∼1 × 1 cm pieces involving the biofilm region of interest (periphery for delaminated buckles, Figure S6B). These pieces were then placed in a muffin silicone mold (with the side involving the region of interest facing the bottom, Figure S7C), and liquid paraffin wax at *T* > 60 °C was poured on top. After 30 min, the excess of solid paraffin was cut with a scalpel. Using liquid wax, the samples were then glued to the sample holders of the vibratome with the side of interest facing up (Figure S7C). A drop of ultrapure water was added to the sample to prevent evaporation. Cuts were performed the same day with a VT1000 S vibratome (Leica, Germany). Thickness was adjusted to obtain 250 μm thick slices, and cross sections were collected with the help of a paint brush or directly floated onto a glass slide for fluorescence imaging.

### Microindentation on Biofilms and Substrate

Biofilms were grown for 4 days and either measured directly after growth or stored in the fridge for less than 5 days. For storage at 4 °C, the Petri dishes were sealed with parafilm to prevent evaporation. 2–4 biofilm samples were tested per condition. Eight measurements were performed in the central region of biofilms, which were still attached to the respective agar substrate. Average and standard deviations were calculated over all measurements from a respective condition. The lateral distance between two measurement points was at least 250 μm in *x* and *y* directions. Microindentation measurements were carried out using a TI 950 Triboindenter instrument (Hysitron Inc.) to determine the load–displacement curves *p*–δ. The instrument was calibrated in air. Indentations were performed with a spherical diamond tip of radius *R* = 50 μm on a stage for the measurement of soft biological samples. The sample surface was approached from 300 to 400 μm above the surface and retracted to the starting position while recording the measured force over the whole range. Loading rates ranged from 20 to 30 μm/s, which translates to loading and unloading times of 10 s. A Hertzian contact model was fitted to the loading part of the curve (indentation depth range δ = 0–10 μm) to obtain the reduced Young’s modulus *E*_r_:^25^
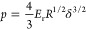
1

Bare agar substrates with all concentrations were prepared in duplicate and tested with the same conditions as biofilms, and data was processed the same way as described above. Agar substrates were prepared 2 days before measurements according to our biofilm growth protocol. The plasticity index ψ is defined as ψ = *A*_1_/(*A*_1_ + *A*_2_), where *A*_1_ describes the area between the loading and unloading curves, and *A*_2_ describes the area under the unloading curve (Supporting Information, Figure S6).

## Supporting Information Available

Rigidity of substrate surface tested by nanoindentation, relative area spreading rates of *E. coli* biofilms, wet and dry gravimetric biomass measurements, bright-field images of cross sections, biofilm thickness measurements, representative load–displacement curve, and an illustration of cross-sectioning protocol (PDF)
