# Supplementary material for: Adaptation of Escherichia coli Biofilm Growth, Morphology, and Mechanical Properties to Substrate Water Content
Source: ACS Biomater Sci Eng. 2021 Oct 21;7(11):5315–25. doi: 10.1021/acsbiomaterials.1c00927 (PMC8579398; doi:10.1021/acsbiomaterials.1c00927)
Supplement: Supplementary file 1 — ab1c00927_si_001.pdf [file ab1c00927_si_001.pdf]

Supporting Information

Adaptation of *E. coli* biofilm growth, morphology and mechanical properties to substrate water content

Ricardo Ziege[1], Anna-Maria Tsirigoni[1], Bastien Large[1], Diego O. Serra[2,3], Kerstin G. Blank[1], Regine Hengge[2], Peter Fratzl[1], Cécile M. Bidan[1*]

[1] Max Planck Institute of Colloids and Interfaces, 14476 Potsdam, Germany

[2] Institut für Biologie/Mikrobiologie, Humboldt-Universität zu Berlin, 10115 Berlin, Germany

[3] Institute of Molecular and Cell Biology, 2000 Rosario, Argentina

**** Corresponding author***

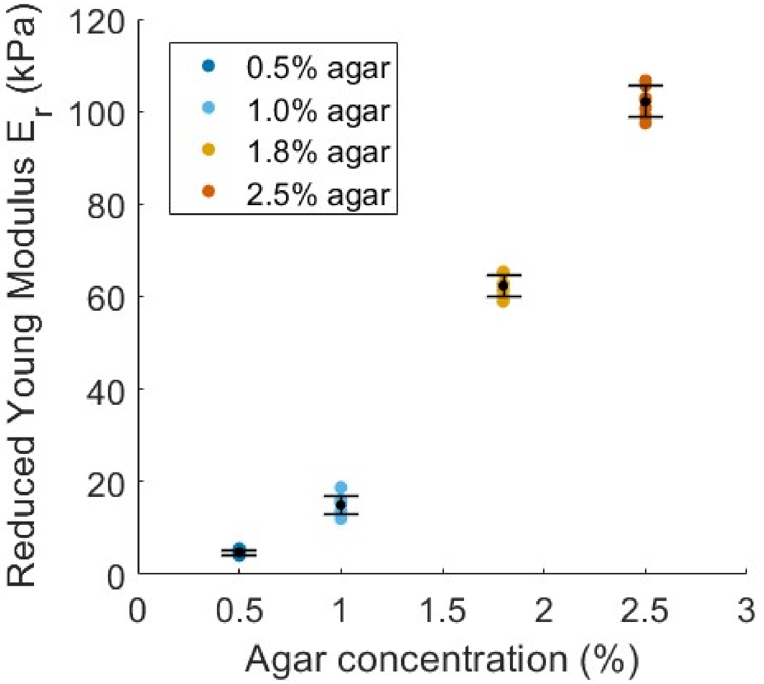

Figure S1: Averaged reduced Young modulus E_r values, describing the measured rigidity of the substrate surface by nanoindentation (from 0.5% to 2.5% agar: 4.8 ± 0.5 kPa, 15.0 ± 2.1 kPa, 62.3 ± 2.4 kPa, 102.1 ± 3.4 kPa; n = 7-8 individual measurements)

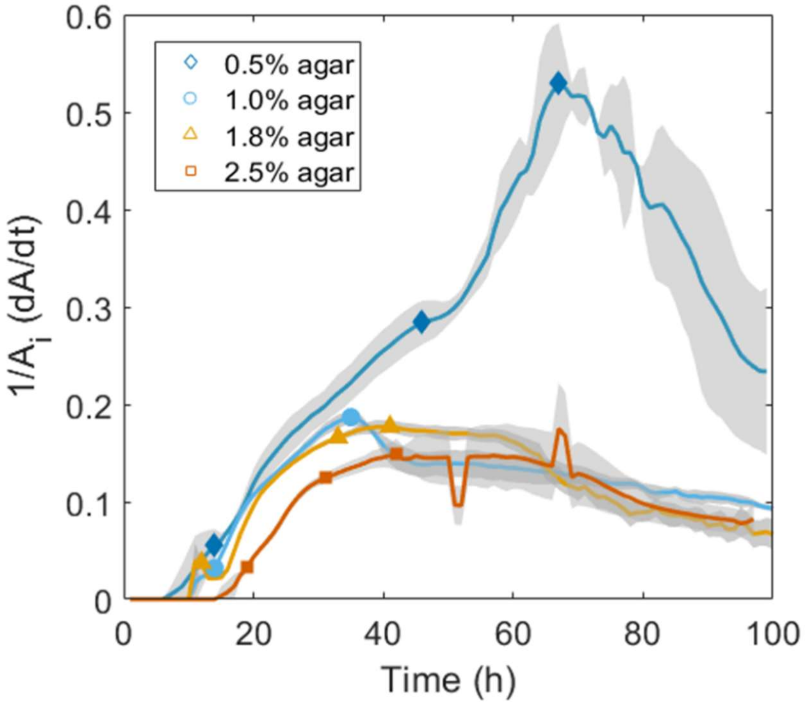

Figure S2: Relative area spreading rates of *E. coli* AR3110 biofilms grown on agar of different agar concentrations, calculated from the derivative $1/A_i$ * dA/dt

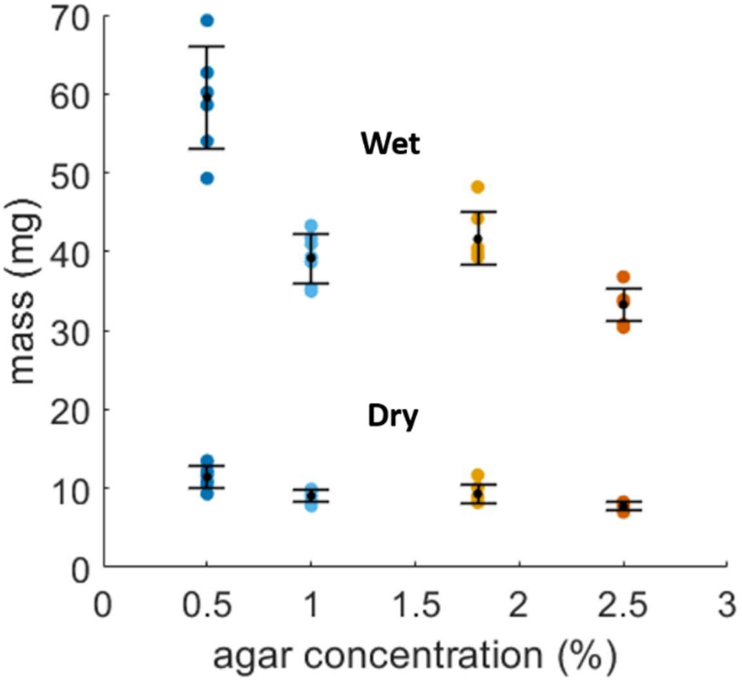

Figure S3: Wet and corresponding dry biofilm mass from gravimetric measurements

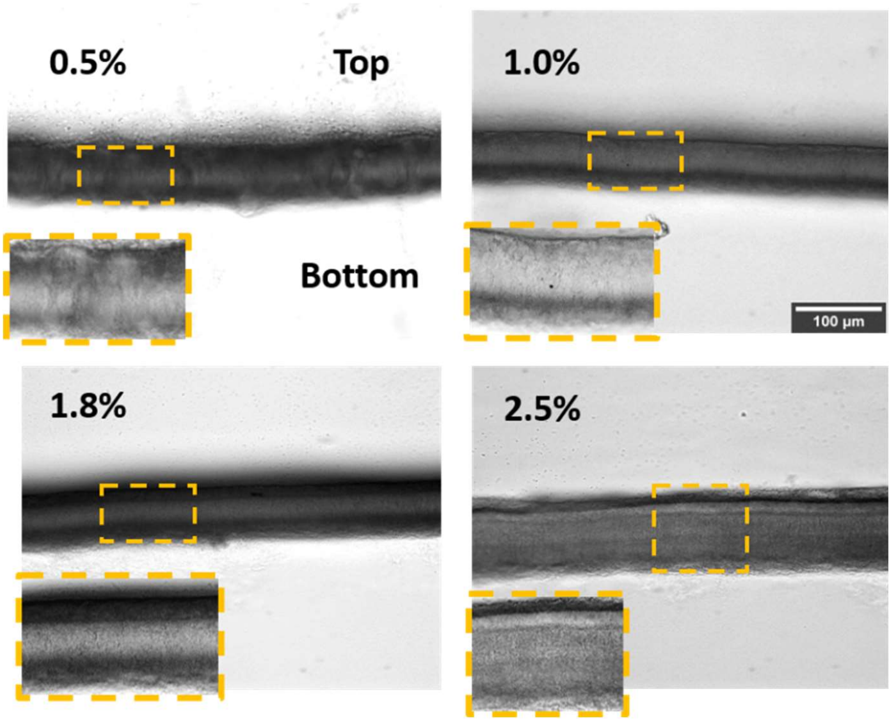

Figure S4: Brightfield images of cross sections corresponding to the fluorescence intensity images shown in Fig. 3C

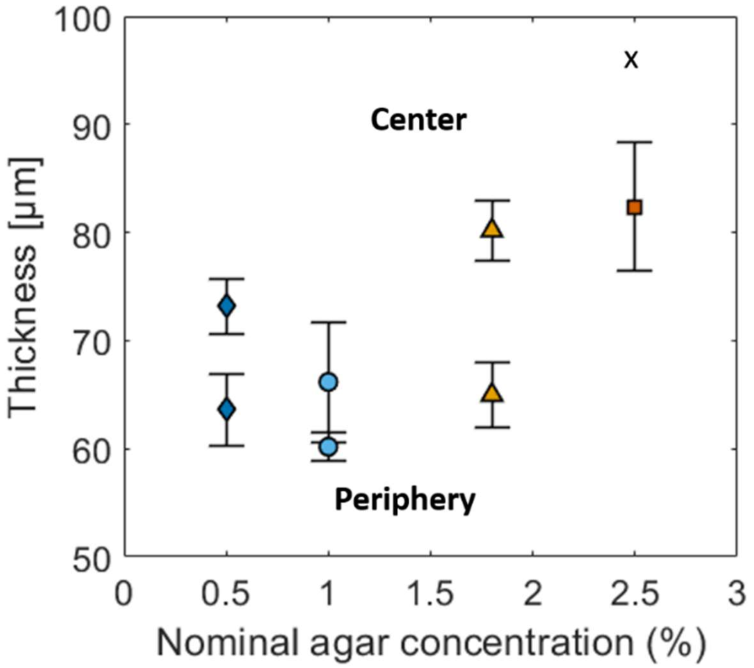

Figure S5: Average biofilm thicknesses (n = 10) measured on brightfield images on central and peripheral biofilm cross sections

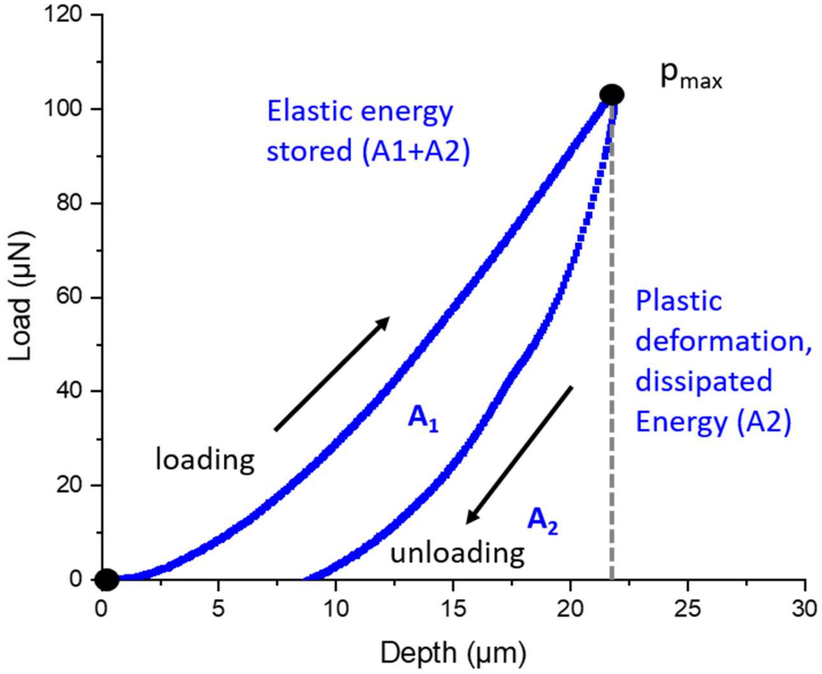

Figure S6: Representative load-displacement curve during loading and unloading AR3110 biofilm surface (1.8% agar). Indicated are the areas used for calculating the plasticity indices (A1, A2).

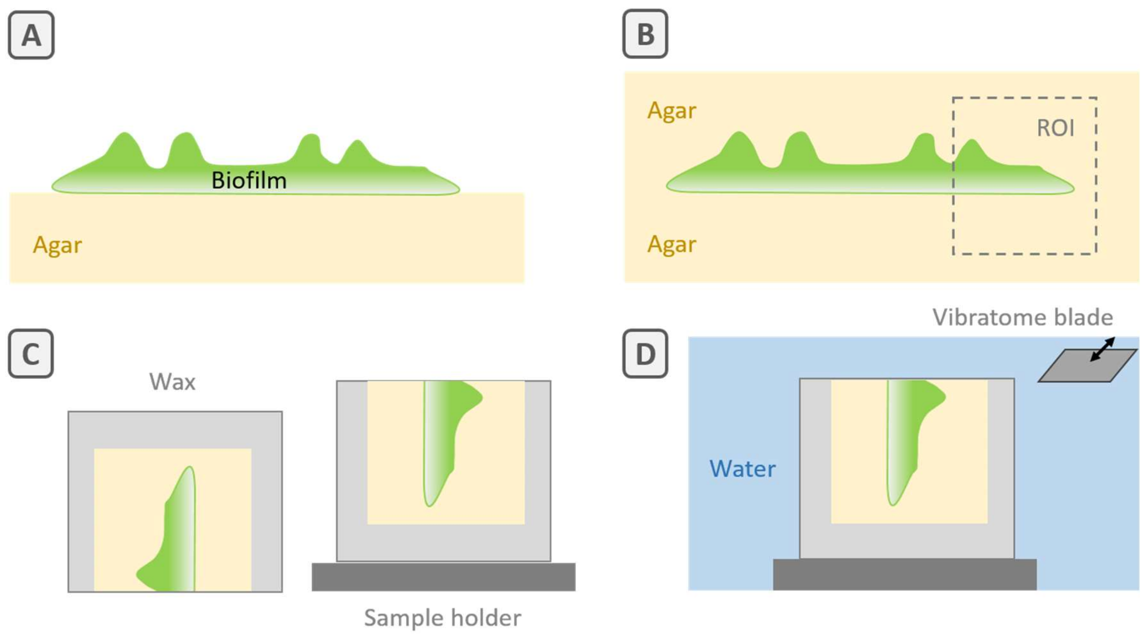

Figure S7: Cross-sectioning protocol of biofilms (A) Isolating of individual biofilms (B) Embedding of biofilms in liquid agar (1.8%) and cutting of region of interest (ROI) (C) Embedding of agar-biofilm-agar sandwich in wax and gluing to the sample holder (D) Performing slices with the VT1000 S vibratome vibrating blade with a lateral distance of 250 µm
